# Insulin-Degrading Enzyme, an Under-Estimated Potential Target to Treat Cancer?

**DOI:** 10.3390/cells11071228

**Published:** 2022-04-05

**Authors:** Laetitia Lesire, Florence Leroux, Rebecca Deprez-Poulain, Benoit Deprez

**Affiliations:** INSERM U1177 Drugs and Molecules for Living Systems, Institut Pasteur de Lille, European Genomic Institute for Diabetes, University of Lille, F-59000 Lille, France; laetitia.lesire@univ-lille.fr (L.L.); florence.leroux@pasteur-lille.fr (F.L.); benoit.deprez@univ-lille.fr (B.D.)

**Keywords:** insulin-degrading enzyme, cancer, target

## Abstract

Insulin-degrading enzyme (IDE) is a multifunctional protease due to the variety of its substrates, its various cellular locations, its conservation between species and its many non-proteolytic functions. Numerous studies have successfully demonstrated its implication in two main therapeutic areas: metabolic and neuronal diseases. In recent years, several reports have underlined the overexpression of this enzyme in different cancers. Still, the exact role of IDE in the physiopathology of cancer remains to be elucidated. Known as the main enzyme responsible for the degradation of insulin, an essential growth factor for healthy cells and cancer cells, IDE has also been shown to behave like a chaperone and interact with the proteasome. The pharmacological modulation of IDE (siRNA, chemical compounds, etc.) has demonstrated interesting results in cancer models. All these results point towards IDE as a potential target in cancer. In this review, we will discuss evidence of links between IDE and cancer development or resistance, IDE’s functions, catalytic or non-catalytic, in the context of cell proliferation, cancer development and the impact of the pharmacomodulation of IDE via cancer therapeutics.

## 1. Introduction

Insulin-degrading enzyme (IDE), also called insulysin, is a ubiquitous 110k Da zinc metalloprotease belonging to the M16 family of metalloproteases. As the name suggests, this enzyme was discovered for its ability to degrade insulin, but it was soon shown to be implicated in the hydrolysis of many other amyloidogenic peptides, such as the amyloid-β peptide IGF-II, glucagon and amylin. Its roles in diabetes mellitus, insulin resistance, and Alzheimer’s disease have been explored for several years and suggest that IDE could be a potential target in these diseases [1,2,3]. IDE is a remarkable enzyme [4] as it (1) is expressed in all tissues, including non-insulin-sensitive tissues, (2) has various subcellular localizations either at the membrane or in the cytosol or various organelles, (3) acts differently depending on the organ and (4) has a unique 3D structure.

Furthermore, IDE is found in many subcellular environments, mainly in the cytosol but also in endosomes [5,6], mitochondria [7], peroxisomes [8], nucleus [7,9] and at the plasma membrane [10]. It is also found extracellularly [11,12,13] and acts as a receptor for the varicella virus [14]. These elements, along with evidence of activities beyond proteolysis, suggest that IDE is a key multifunctional protein [15]. 

In addition to the physiological and physiopathological roles of IDE in the diseases mentioned above, we review here the potential roles of IDE in cancer via both substrate hydrolysis activities and other functions.

## 2. An Atypical Structure at the Basis of IDE’s Multiple Roles

IDE displays two homologous 50 kDa domains joined by a short hinge loop, defining a large inner chamber [16]. One domain contains the conserved HXXEH zinc ion-binding motif [17]. IDE adopts two conformations, an open conformation that allows internalization of the substrate and release of the products and a closed conformation that reconstitutes the catalytic site for the substrate hydrolysis. The displacement of a subdomain called a “swinging door” unveils an opening towards the enclosed catalytic chamber, which has been recently evidenced by both X-Ray and CryoEM [18,19].

**Figure 1:**
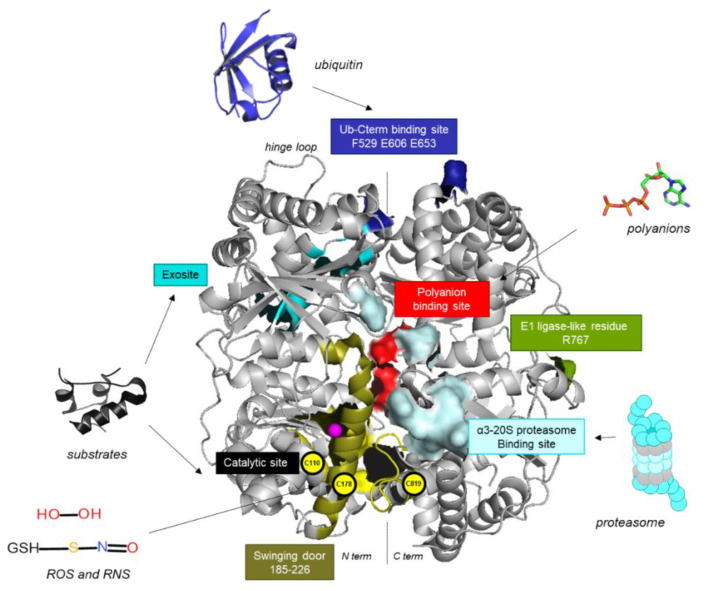
Structure of IDE (from 4NXO). Catalytic site in black; Zn2^+^ ion in magenta; swinging door in olive exosite in cyan [19]; 20S proteasome binding in light blue [27]; E1 ligase-like activity important residue in green [29]; Ub-C-term binding in marine [28]; polyanion binding site in red [25]; cysteins sensitive to reactive oxygen species (ROS) or reactive nitrogen species (RNS) in yellow spheres [30].

X-Ray crystallography and SAXS studies have revealed that IDE can form a dimer. This dimer also undergoes an open-to-closed transition promoted by substrate binding [19]. IDE uses its catalytic chamber to trigger the unfolding of substrates, as proven by X-ray structures that show the substrates in a partially unfolded state inside IDE [20]. IDE uses the size and the electrostatic properties of the catalytic chamber to selectively bind peptides that are less than 80 amino acids in length and have a high dipole moment (Figure 1). Hydrophobic and aromatic residues of the active site are essential for peptide hydrolysis [21].

IDE is found and evolutionarily conserved in a vast number of eukaryotes, including plants and lower organisms such as yeast, even though most of them do not produce cognate substrates, suggesting a key role in cell biology yet to be fully understood. In particular, IDE has a well-conserved exosite (Figure 1). By binding to the N-terminal end of IDE substrates, this exosite facilitates the unfolding of substrates [22], allowing multiple cleavage sites by IDE. Additionally, the binding of short substrates to the exosite seems to play a regulatory role by reducing the size of the IDE catalytic chamber and thus entropically favors the binding of a second molecule of the substrate at the catalytic site [23]. Additionally, IDE has an allosteric regulatory site at IDE-C, where ATP attaches and selectively accelerates the degradation of the short peptides [24,25,26] (Figure 1).

Several important studies have also identified key amino acids for IDE interactions with an α3-20S proteasome subunit (residues R674; R782; F486; E962; E964) [27] or ubiquitine C-term (residues F529 E606 E653) [28].

## 3. A Long List of Substrates

IDE behaves primarily as a proteolytic enzyme involved in the degradation of many substrates, amongst which several are of interest in cancer biology (Table 1).

IDE is the main enzyme responsible for the intracellular hydrolysis of insulin [31], glucagon [33], and amylin [61]. Other peptidic hormones secreted by the pancreas are also substrates of IDE. Of note, however, is the large difference in Km between insulin, on the one hand, and glucagon and amylin, on the other hand. This questions the biological relevance of IDE-mediated hydrolysis of glucagon and amylin, as no proof of involvement of IDE in the clearance of these peptides in vivo exists. Most of the reported Km are far above normal plasma concentrations of these substrates, even for insulin which peaks at 2nM, roughly 30 times lower than the Km. However, there is indirect proof of the involvement of IDE in insulin degradation. Indeed, pharmacological inhibition of IDE increases insulin concentration in plasma and insulin signaling in target organs [62]. To interpret the biological relevance of IDE activity in degrading its substrates, it is still necessary to measure their actual concentrations, possibly in all possible subcellular locations where both substrate and IDE are found, and compare them with their Km. In 1994, studies in rats by Kurochkin et al. showed that IDE interacts with radiolabeled Aβ in both the liver and brain [57]. IDE was then proposed as one of the enzymes involved in the catabolism of Aβ. Hydrolysis of CGRP by IDE was evidenced in spinal cord lysates of mice [45], and this hydrolytic clearance was shown to be entirely IDE-dependent. Many other peptides with various lengths and affinities have been reported as substrates of IDE-like somatostatin [35], IGF-2 [47], TGF-α [49], β-endorphin [41], dynorphins [41], bradykinin [23], atrial natriuretic peptide (ANP) [37], chemokine ligand (CCL)3 and CCL4 [52,53] and ubiquitin [55]. The involvement of IDE in the degradation of all these substrates suggests its potential role in processes modulated by these peptides. Interestingly, most substrates of IDE share a propensity to be amyloidogenic peptides [63], with a few exceptions like HIV-1 p6 [64].

IDE has been thus shown to hydrolyze more than 15 different substrates that are involved in cancer pathophysiology. More specifically, insulin, IGF-2 and CCL3 will be detailed below.

## 4. IDE: A Chaperone-Like Protein?

More and more observations point towards the functions of IDE in cells beyond its catalytic activity. In particular, many studies have demonstrated interactions with some key proteins and have suggested a chaperone-like activity of IDE. IDE is indeed overexpressed in different human cell lines, cancerous and non-cancerous, after environmental stress such as heat shock, oxidative stress and serum starvation, parallel to HSP70. This suggests that IDE could be a heat shock protein. The chaperone effect of IDE is supported by the presence of heat shock elements (HSE) in the IDE gene promotor and by the presence of high-affinity binding sites for the heat shock factor [54]. Moreover, it was observed that during a heat shock response, newly synthesized IDE is translocated from the cytosol toward the ER, an important sub-cellular compartment for protein folding [65,66]. In the viral context of VZV, IDE bound the non-glycosylated precursor of glycoprotein E in the endoplasmic reticulum [67]. In particular, recombinant soluble IDE is able to induce a conformational change of the glycoprotein E that enhances the infectivity and stability of the virus [68]. Some authors have described IDE as a “dead-end” chaperone that forms highly stable complexes with amyloid peptides Aβ and α-synuclein [69,70]. Independently, it was also observed that IDE is able to block the amyloid pathway by promoting non-fibrillar aggregate formation in a non-catalytic way [70,71]. Interestingly, α-synuclein has a dual effect in cancer by promoting tumorigenicity and inhibiting cancer growth according to the models studied in the different studies [72]. Ramaraju et al. describe the kinetic model of two IDE-bound states, one used for proteolysis and the other as a kinetic trap to differentiate non-amyloidogenic and amyloidogenic substrates [64]. Finally, IDE interacts with intermediate filaments, vimentin and nestin complexes during mitosis. These interactions modulate its catalytic activity in a substrate-dependent manner [73]. Vimentin and nestin play an active role in tumorigenesis, particularly in metastasis and tumor growth [74].

## 5. IDE’s Close Link with the Proteasome

The ubiquitin-proteasome system (UPS) and autophagy are the two main pathways for cell protein degradation [75,76]. Several studies show a strong functional link between IDE and UPS. In particular, ubiquitin has been shown by Ralat et al. to bind to IDE and to behave as a substrate, with several amide bonds being sequentially hydrolyzed, starting with the cleavage of two C-terminal glycines [55,77,78]. Conversely, Grasso et al. show that IDE can form K48 and K63 ubiquitin dimers like an E1-like ubiquitin-activating enzyme. However, IDE does not assemble poly-ubiquitin chains [28,29]. Besides the direct observations of ubiquitin dimerization and catalytic hydrolysis of ubiquitin, another study by Tundo et al. shows that transfection of SY5Y cells with an IDE siRNA reduces the amount of poly-ubiquitinylated proteins [65]. The modulation of the proteasome could also explain this last observation regarding IDE. Indeed, IDE interacts with the 26S and 20S proteasomes and behaves as a competitor of the 19S proteasome to bind to 20S [27,65,79]. The activity of the 20S proteasome is then impacted by IDE in a bimodal manner, as explained by the existence of two binding sites displaying different affinities for IDE. The binding of IDE to the site of high affinity (13 nM) is consistent with the hypothesis that IDE could efficiently modulate h20S gating mechanisms [27]. Moreover, transfection of SHSY5Y cells with an IDE siRNA increases all three activities of the 26S proteasome, chymotrypsin, trypsin and caspase, and this modulation does not involve IDE catalytic activity [66]. Fawcett et al. showed the inhibition of the proteasome by insulin. The binding of insulin to IDE would limit the activating interaction between IDE and the low-affinity binding site on the 20S proteasome [79,80].

The proteasome is an important therapeutic target that led to the development of bortezomib almost twenty years ago, followed by other inhibitors (carfilzomib, ixazomib, oprozomib) in a variety of hematologic malignancies [81]. In this context, a pharmacological intervention targeting IDE could possibly increase the efficacy and/or the potency of antiproliferative activity of proteasome inhibitors.

## 6. Expression of IDE in Human Cancers

The first protein expression of IDE was measured by cell microarray in more than thirty human tumor cell lines by Schmitt et al. Expression of IDE was confirmed in all tested lines from solid and blood tumors, except two cell lines (Raji lymphoma cells and HL-60 leukemia cells) [82,83]. More interestingly, overexpression of IDE was observed by immunochemistry in malignant human breast cells [84]. In the same way, Tundo et al. showed by immunochemistry that IDE is overexpressed in tumors of the central nervous system (similarly high in grade III and IV malignant glioma cells and olfactory neuroblastoma tumor cells) in comparison to normal nerve cells, suggesting a role of IDE in tumor progression [65]. Recently, in a study of the expression of genes related to the insulin and inflammatory pathways in breast cancers, IDE overexpression appears to be a risk factor for relapse and contributes to disease-free survival [85]. In another breast cancer cohort, IDE protein content in the cytoplasm of cells increased in the tumor compared to the normal mammary gland [84]. In the latter study, a higher proportion of loss of heterozygosity in the locus harboring the IDE gene was found in high-grade tumors. Despite the variety of modalities used to measure differences in IDE expression in disease tissues versus controls (mRNA or protein content using antibodies), these studies point out IDE as a potential pharmacological target to treat cancer.

## 7. IDE and the Insulin/Insulin-Growth Factor Signaling (IIS) Pathway

Insulin and IGF, two genuine substrates of IDE, control critical pathways in energy metabolism and growth, especially for cancer cells [86,87,88]. Dietary and lifestyle factors that impact this pathway are identified as cancer risk factors. In line with this observation, obesity is a cancer risk factor mediated by insulin resistance, hyperinsulinemia, adipokines secretion by adipose tissue, increasing IGF expression, and chronic inflammation [89]. The International Agency for Research on Cancer (IARC) working group reported from meta-analyses or pooled analyses that the relative risk of cancer for obese people was 1.5 to 1.8 for colon, gastric cardia, liver, gallbladder, pancreas and kidney cancers [90]. In addition to being a risk factor, in a prospective cohort study, people with a body mass index superior to 40 had a 52% (men) and 62% (women) higher risk of death from all cancer than people with normal weight [91]. In the same way, type 2 diabetes, dyslipidemia, hyperglycemia, and metabolic syndrome also constitute cancer risk factors [92,93].

**Figure 2:**
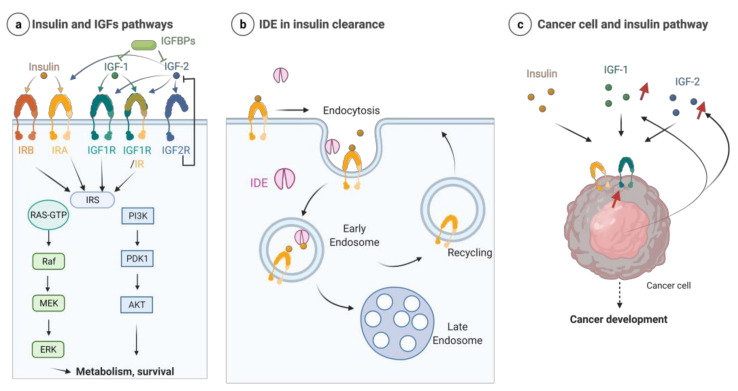
(**a**) Condensed representation of the insulin and IGF pathways. After ligand binding and activation, insulin and IGF1 receptors induce a signal through IRS via MAPK and AKT pathways to control cell metabolism and survival. IGFBP proteins bind IGFs and reduce their signals. The binding of IGF2 to IGF2-R leads to internalization and lysosomal degradation. (**b**) IDE is involved in insulin clearance, particularly in endosomes, after activating insulin receptors by its ligand. (**c**) Cancer cells overexpress insulin and IGF1 receptors. Cancer cells can produce IGF proteins that act in an autocrine and paracrine manner. Insulin and IGF pathways contribute to cancer development. Red arrows mean overexpression. Created with BioRender.com (accessed on 31 March 2022).

Insulin binds two receptors to the isoforms insulin receptor A (IRA) and B (IRB). IGF-I binds the IGF1 receptor (IGF1-R) or the hybrid IR/IGF1-R, while IGF-II binds IRA, IGF1R, the hybrid IR-IGF1R and the IGF2 receptor (IGF2-R). For the latter, ligand binding is followed by internalization, lysosomal degradation and downregulation of IGF2. When bound to an agonist, all other receptors transduce a signal with IRS (insulin receptor substrate), which activates the MAPKinase and PI3K/AKT pathways. IGFBP (IGF binding proteins) bind the IGF and, in most cases, limit its activities (Figure 2a).

IDE has been shown to play a significant role in the downstream signaling of insulin. It is involved in insulin clearance in the extracellular compartment and at the plasma membrane, the endosome, and the cytosol. Receptor-bound insulin is internalized into early endosomes where IDE can hydrolyze insulin in this non-acidic environment. The recruitment of IDE in endosomes can be promoted through phosphatidylinositol phosphates binding at its polyanion site [6]. In the acid late endosome, insulin is dissociated from its receptor and is degraded; the role of IDE in this context is discussed [6]. Then, the insulin receptor can be recycled back to the plasma membrane [94,95] (Figure 2b). 

While Miller et al., in a rodent model of *Ide* deficiency, failed to detect hyperinsulinemia [96], Farris et al. showed that *Ide* gene ablation in mice reduces insulin clearance and induces glucose intolerance and moderate hyperinsulinemia [97]. Recently, specific deletion of the *Ide* gene in the mouse liver did not show hyperinsulinemia as expected but an impairment of insulin signaling by downregulating the insulin receptor expression at the plasma membrane [98]. This study suggests a more complex role of IDE in both insulin clearance and trafficking. The transmembrane glycoprotein CEACAM1 (carcinoembryonic antigen-related cell adhesion molecule 1) is involved in the endocytosis of the insulin receptor activated by insulin. Najjar et al. proposed a cooperative role between IDE and CEACAM1 for insulin trafficking [99]. The ambiguous role of CEACAM1 in human malignancies, reported both as a tumor suppressor or as a tumor progression, angiogenesis, and immune evasion factor, depending on malignancies, could be explained by variations in IDE expression or catalytic activity in different pathological settings [100].

While IDE regulates both insulin level and signaling in multiple ways, insulin, in return, appears to regulate IDE expression. Indeed, in hippocampal neurons, insulin upregulates the expression of IDE through the PI3K/AKT pathway [101]. However, in HepG2 cells, a 24 h treatment with insulin increases IDE activity without changing its expression (mRNA and protein levels), and this modulation is affected by glucose concentration [102]. These findings suggest that insulin signaling modulates IDE expression and/or activity depending on cell type and environment. 

Cancer cells express insulin and IGF receptors. Several studies demonstrated overexpression of insulin receptors (IR), preferentially IR-A, and IGF1 receptors (IGF1-R) in malignant cells [103,104]. Unlike insulin secreted exclusively by pancreatic β cells, IGFs, which are produced mainly by the liver, can also be produced by cancer tissues and act through paracrine and autocrine mechanisms [105]. Insulin and IGFs participate in the pathogenesis, progression and prognosis of cancer by increasing cancer cell proliferation (Figure 2c). In this way, preclinical studies of drugs that target IGF1 and/or insulin pathways suggest that modulating this pathway could find applications in cancer [86]. An insulin signature based on the differential expression of 15 genes has been observed in breast cancer and associated with 8-year disease-free survival. In this list, overexpression of IDE constitutes a high-risk factor [85]. Some cancerous animal models displayed a decrease in systemic insulin due, in part, to high amounts of IDE released by tumor cells. In this case, treatment of mice with insulin improves the cardiomyopathy associated with these models and decreases tumor growth [106].

At last, the metabolism of cancer cells is characterized by the Warburg effect, which consists of aerobic glycolysis and the production of lactate. It has been described that in liver lysates, L-lactate indirectly regulates IDE activity [107]. It would be interesting to know how IDE is modulated by cancer cell metabolism.

In all, how insulin modulates IDE expression levels or activity in cancer cells in different models, with and without the microenvironment, and how IDE inhibition in cancer cells affects the insulin/IGF pathways and, consequently, tumor metabolism and survival, are key questions worth exploring.

## 8. IDE and Sex Hormones

Besides metabolic control of cell growth by insulin and IGFs, some tumors such as prostate or breast cancer are driven by sex hormones. Several teams have reported a close link between IDE expression and sex hormones, even though the molecular mechanisms underlying this interplay and its functional consequences remain elusive. Indeed, estrogen can induce IDE expression in a brain-region-specific manner [108]. Another study demonstrates that testosterone and estrogen upregulate IDE expression in rat prostate and uterus [109]. An increase in IDE expression has been observed after dexamethasone plus testosterone treatment in castrated rats, suggesting an important role of IDE in tissue remodeling [110]. At the least, IDE enhances the DNA binding of androgen and glucocorticoid receptors, but the consequences of these interactions, notably on transcription, remain to be described in detail [9]. Therefore, the link between sex hormones and IDE demonstrated so far deserves further studies in the field of cancer.

## 9. IDE, Tumor Suppression and Proliferation

Cancer cells result from genetic alterations that activate oncogenes and/or inactivate tumor suppressor genes. Among the latter, retinoblastoma protein (Rb) is a tumor suppressor which inhibits cell cycle progression by interacting with E2F transcription factors. A study by Radulescu et al. shows that IDE can be co-purified with Rb from proteasomal preparation in two cancer cell lines [111]. They hypothesized that IDE could protect Rb from inactivation by insulin [111,112].

Another tumor suppressor that interplays with IDE is PTEN (phosphatase and tensin homolog deleted from chromosome 10). PTEN inhibits cell growth by antagonizing the PI3K-AKT-mTOR pathway and, as such, is frequently mutated and inactive in cancers. A study demonstrated that under nutritional starvation, IDE and SIRT4 contributed to PTEN degradation, inducing increased autophagy and cell survival [113]. In this study, knockout of IDE in cancer cells had a minimal effect on cell cycle profile, cell migration and cell growth, but upon serum starvation, the absence of IDE decreased proliferation and migration. In the same way, the knockdown of *Ide* in neuroblastoma cells also decreased cell proliferation and induced cell apoptosis [65]. The same observation was made in HepG2 cells, where IDE knockdown decreased cell proliferation [114].

## 10. IDE, Tumor Microenvironment and Stress

**Figure 3:**
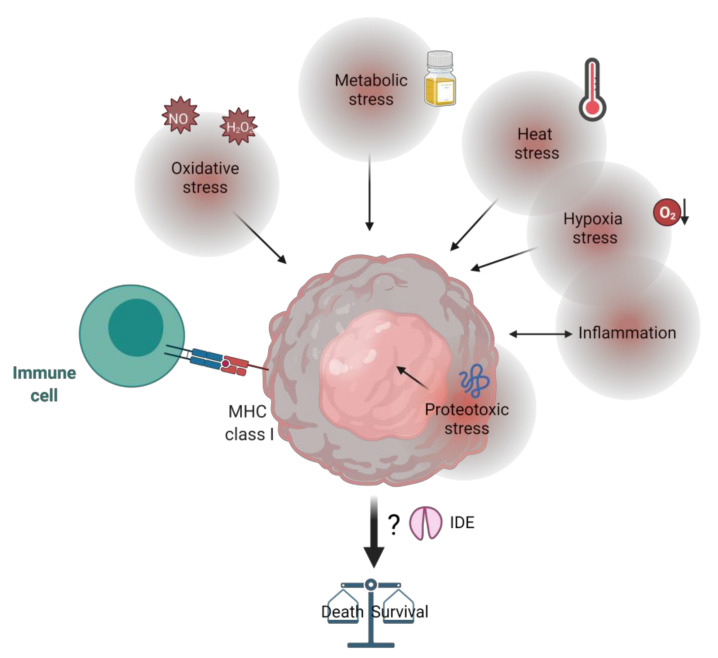
Impact of the environment on cancer cells that could involve IDE modulation as a response. Cancer cells are submitted to various stresses such as oxidative, metabolic, heat, hypoxia, proteotoxic stresses, inflammation and immune recognition that induce a response that can allow cell survival or death. It is known that IDE can modulate or be modulated by these different stresses. Created with BioRender.com (31 March 2022).

Cancer cells are submitted to various environmental stresses that force them to adapt. Interestingly, IDE expression and functions are sensitive to environmental stress, including oxidative stress, serum starvation, proteotoxic stress, and heat stress [65,113,115] (Figure 3). It is hypothesized that IDE improves cell survival under stress conditions, a context highly relevant to tumor cells. In this way, IDE appears to play a role in the cell response to endoplasmic reticulum stress, referred to as the unfolded protein response (UPR). The UPR is an adaptive response to the accumulation of unfolded proteins in the ER that can restore cell homeostasis and drive cell death if the stress is not rapidly resolved. This natural balance is involved in normal and pathological situations, particularly in cancer, where cancer cells need to adapt and survive under stressful environmental conditions [116]. Minchenko et al. have recently shown that IDE expression is modulated by the IRE1α sensor of the UPR pathway in glioma cells [115]. The IDE homologue Iph1 in yeast participates in the ER stress response induced by tunicamycin [117]. In line with these observations, a potential binding site for XBP1 and HIF transcription factors in the IDE promoter region has been identified [115]. 

Autophagy, a catabolic process that involves the formation of autophagosomes and the lysosomal degradation of proteins, is another response to intrinsic or extrinsic stresses. Because it is involved in cancer pathogenesis, the modulation of autophagy has been proposed as a cancer therapy [118]. Interestingly, it was demonstrated that IDE secretion increases with autophagy flux in astrocytes [12,119]. However, the link between IDE and autophagy in a tumor context and the relevance of increased IDE secretion in this context have not yet been studied.

Additionally, cancer cells can escape recognition and destruction by T-cells. In this way, IDE could impact the antigen presentation on major histocompatibility complex (MHC) class I molecules independently of the proteasome. Indeed, IDE can process a tumor protein, MAGE-A3, and participate in cytotoxic T lymphocyte recognition of tumor cells [120]. In another study, IDE did not affect presentation on five epitopes from ovalbumin, the envelope protein of HIV, type 1 diabetes autoantigen IGRP, proinsulin and beta-amyloid, or MHC class one expression [121]. Therefore, it seems that the role of IDE in antigen presentation remains only occasional. However, since IDE is a peptide processing protease, its role in antigen presentation, specifically in cancer, warrants further study [122].

Inflammation, whether extrinsic or intrinsic, is often associated with tumorigenesis [123]. In this way, CCL3 (also known as macrophage inflammatory protein-1α, i.e., MIP-1α) and CCL4 (MIP-1β) are pro-inflammatory chemokines that display both pro-and anti-cancer properties. On the one hand, they cause migration and invasion of cancer cells, angiogenesis and lymphangiogenesis. On the other hand, they have anti-cancer properties by recruiting anti-cancer tumor-infiltrating lymphocytes [54]. Interestingly it was shown that IDE degrades monomeric CCL3 and CCL4, reducing the chemotactic activity of these cytokines [52,53]. Additionally, IL-6 in HepG2 and C2C12 cells increases IDE expression and activity [124]. A recently described IDE peptidic inhibitor reduces the pro-inflammatory Th17 response against insulin in the NOD mouse model, supposedly by impacting the inflammatory responses of CD4+ T-cells toward the insulin beta chain peptide, a major epitope for T-cell activation [125]. These observations create an additional link between the protease and regulation of the tumor environment.

Caravaggio et al. also reported that IDE could associate with the cytoplasmic domain of the macrophage scavenger receptor-A (SR-A, CD-204) [126] and studied this in the context of foam cell formation in atherosclerosis. Still, this interaction between SR-A and IDE may be highly relevant in the field of oncology, as SR-A-dependent macrophage functions have been reported in endoplasmic reticulum stress-induced autophagy in macrophages [127], in tumor progression in ovarian and pancreatic cancer [128], as a marker of prognostic in prostate cancer [129], directly involved in tumour infiltration by the immune microenvironment and in the progression of colorectal cancer [130].

P-glycoprotein (P-gp) or MDR1 (multidrug resistance protein 1) expressed by cancer cells contribute to drug resistance [131]. In three cancer cell lines, it was shown that IDE interacts with this efflux pump [132]. However, the consequences of this interaction are not known, particularly for drug resistance, and need further exploration. 

Additionally, IDE displays 13 cysteine residues. Its activity and oligomerization were shown to be sensitive to oxidation (H_2_O_2_) and nitrosylation (S-nitrosoglutathione) [30]. The cysteines in IDE act as sensors of reactive oxygen species and reactive nitrogen species. The modification of Cys-819 or Cys-110 leads to the loss of enzymatic activity, while the modification of Cys-178 is protective and prevents both IDE inactivation and oligomerization. The impact of these modifications was also shown to be substrate-dependent [30]. Reactive oxygen and nitrogen species can have many effects, including the post-translational modification of many proteins at critical cysteine thiols. This is highly relevant in the context of cancer, where both S-oxidation and S-nitrosylation of proteins occur under the tumor environment and impact cancer cell proliferation and even drug resistance [133,134]. In that context, IDE could be both a target for these events and a sensor for ROS and nitric oxide within cells.

## 11. IDE Is a Druggable Target

Importantly, IDE can be considered a druggable target for the following reasons: (i) in vivo knockout mice have been described and characterized and allow the comparison of the effect of pharmacological modulators with a wild type environment; (ii) numerous inhibitors from chemically diverse families have been disclosed; (iii) target-engagement tools have been developed; (iv) several crystal structures of IDE with or without ligands are available to rationalize the structural impact of ligand binding. Notably, IDE’s two different enzymatic activities, namely the proteolytic activity and the ubiquitin-activating E1-like activity, can be differentially targeted by inhibitors. Indeed Bellia et al. reported in 2019 that Copper II inhibits the proteolytic activity of IDE without compromising the ubiquitin ligase activity [29]. This is consistent with the fact that these activities occur in different subsites of the protein.

Several models of *Ide*−/− mice or tissue-selective *Ide*−/− mice have been disclosed and studied, especially regarding metabolic or Aβ degradation phenotypes. These have significantly contributed to exploring the causal implication of IDE, respectively, in type 2 diabetes and Alzheimer’s disease. For example, Farris et al. reported for the first time that *Ide*−/− mice display hyperinsulinemia and glucose intolerance, as evidenced by hyperglycemia after IGTT compared to control mice [97]. This phenotype, including insulin resistance, hyperinsulinemia, glucose intolerance, and increased weight, appears to be age-dependent [135]. In *Ide*−/− mice, where expression of neprilysin (the other enzyme responsible for Aβ clearance) was conserved, the levels of Aβ are enhanced by 65% [97]. GK rats, which have a loss-of-function IDE mutation, exhibit symptoms of type 2 diabetes and impaired degradation of Aβ [136].

Recent tissue-selective deletion of *Ide*, in β-pancreatic cells [137], liver cells [98] or bone marrow [126] in mice has allowed the role(s) of IDE in metabolic phenotypes like β-cell function, insulin resistance, glucose tolerance, insulin clearance or atherosclerosis to be refined. All these models could be of high value for the exploration of IDE implications in cancer phenotypes.

Several IDE modulators, mainly inhibitors, have been developed in the past decade and could be used to chemically validate IDE in different phenotypes related to cancer. These compounds display various behaviours regarding substrates and different drug-likeness properties, allowing us to explore the various functions of IDE both in cells and in vivo.

Both small organic compounds and peptidic and pseudopeptidic compounds have been disclosed. While most inhibitors of IDE are pan-substrate inhibitors (**BDM44768, Ii1**) [62,138], some are selective for a single substrate [139] (**6bK**) or behave as activators for some substrates (**BDM43079**) [140,141]. These small-organic or peptidic compounds were most often discovered either by the screening of diverse or focused libraries [140,141], DNA-templated libraries (**6bK)** [139], fragments [142] or drugs [143] (**ebselen**) or discovered via a kinetic target-guided synthesis (**BDM44768**) [62]. A few inhibitors were designed by rational design, taking inspiration from either insulin (**Ii1**) [138] or a VZV peptide (compound **ADT21).** A recent beta-hairpin inhibitor of IDE, **B35**, was designed as a constrained mimic of the insulin B chain binding sequence EALYLVCG [144]. [Pt(O, O’-acac)(γ-acac)(DMSO)] was disclosed as a novel inhibitor of IDE [145]. Interestingly, it was evaluated on neuroblastoma cells, in which IDE overexpression has been linked to more aggressive tumors and progression, proliferation and viability. This new inhibitor decreased the viability of these cells [145].

Fewer activators are known for IDE. Several of them were discovered by screening. Cabrol et al. discovered the activators Ia1-2 [146]. Recently, Kraupner et al. reported an ATP binding site activator (BDM35899) [140,141]. To our knowledge, however, none of the IDE activators have yet been used in vivo.

Along with inhibitors, methods to evaluate target engagement by these compounds [147] were developed in HegpG2 cancer cells.

Finally, the availability of 3D structures of IDE obtained via X-ray diffraction of the apoprotein or the liganded protein or via CryoEM has helped to understand the binding of inhibitors as well as to rationalize their development. Inhibitors were shown to bind either the catalytic site (**BDM44768, Ii1**) or the exosite (**6bK**) or both (**BDM43079**). A recent study using CryoEM has elucidated how various families of inhibitors disturb the disordered IDE catalytic cleft and how their binding impacts the dynamics of the door subdomain of the enzyme, thus modifying the catalytic activity [19]. Along with the activity of the modulators, these structural data could help rationalize which of the roles of IDE in cancer depend on its catalytic activity or come from another function.

## 12. Conclusions

**Figure 4:**
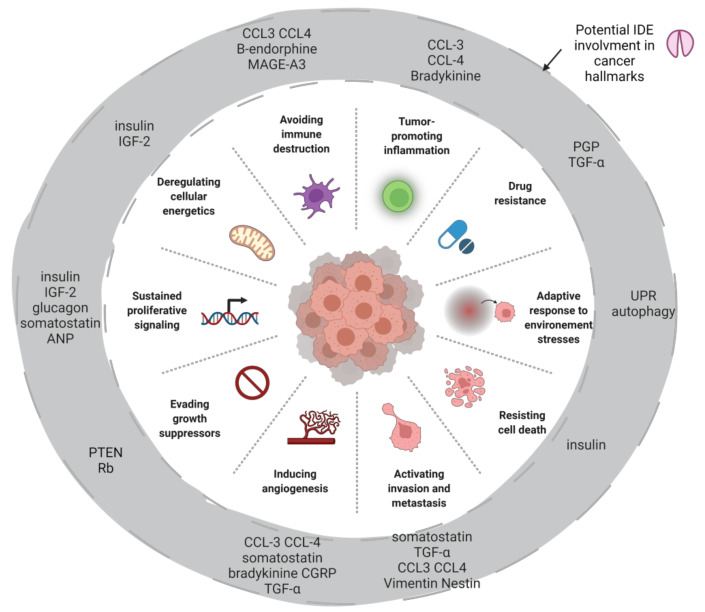
Potential IDE involvement in some cancer hallmarks by its substrates, its protein-protein interactions or by itself with its modulations. The main effect of substrates is reported and can act positively or negatively on cancer hallmarks (Table 1). Created with BioRender.com (31 March 2022).

IDE has been shown to be overexpressed in some cancers. Numerous IDE substrates are highly relevant in the context of cancer, in particular IGF-II, insulin and CCL3. Aside from its proteolytic activity, IDE was shown to interact closely with the ubiquitin-proteasome system and display chaperone or E1-ligase-like activities. IDE has been implicated in the proliferation and survival of cancer cells. Other studies have highlighted how its expression or activity impacts and is impacted by the tumor microenvironment. There is now a body of evidence that IDE is implicated in several hallmarks of cancer (Figure 4) that are worth exploring in a tissue-dependent and substrate-dependent manner.

Interestingly, thanks to the availability of a large variety of IDE modulators as well as KO animals, it is relatively straightforward to engage in comprehensive pharmacological studies to understand IDE’s role(s) in cancer and generate key data for the qualification of this protease as a target in oncology both in vitro and in vivo. Indeed, several IDE modulators initially evaluated in vivo mostly for their effect on glucose metabolism display good animal exposure, suggesting that they can also be used in vivo to explore IDE implications in cancer.

## Figures and Tables

**Table 1:** Properties of main substrates of IDE involved in cancer.

Substrates	Substrate Characterization				Cancer Effect ^1^		
	Length (Residues)	Affinity (Km in µM)	PDB Codes	References	Pro	Anti	Ref.
Hormones							
Insulin	51	0.070	2G54; 2G56; 2WBY; 6BF8; 6BFC; 6B3Q	[31]	X		[32]
Glucagon	29	3.5	2G49	[33]	X		[34]
Somatostatin	14/28	7.5	-	[35]		X	[36]
ANP	28	*nd*	3N57	[37]		X	[38,39]
CNP	22	*nd*	-	[40]		X	[38,39]
Neuropeptides							
Β-endorphin	31	13	-	[41]	X	X	[42]
Dynorphin (B9)	9	27	-	[41]		X	[43]
Bradykinin	9	4200	3CWW	[23]	X		[44]
CGRP	37	nd	-	[45]	X		[46]
Growth Factors							
IGF-2	67	*nd*	3E4Z	[47]	X		[48]
TGF-α	50	*nd*	3E50	[49,50]	X		[51]
Cytokines							
CCL3	70	*nd*	3H44	[52,53]	X	X	[54]
CCL4	69	*nd*	4RAL	[52,53]	X	X	[54]
Others							
Ubiquitin	76	*nd*	3OFI	[55]	X ^2^		[56]
Amyloid β	40–42	2	2G47; 2WK3	[57]		X	[58,59,60]

^1^ Main effect of the substrate in cancer described in the literature. Pro and anti-tumor effects, direct or indirect, can include all stages of cancer initiation, promotion and propagation. References refer to a general review on the role of the substrate in cancer or if it is not available in an article. ^2^ Perturbation of ubiquitination system in cancer; the effect depends on substrates. *Nd*: not determined.

## Data Availability

Not applicable.
